# Multi-omics analysis reveals *Parabacteroides distasonis* and *Kineothrix alysoides* as potential key bacterial species associated with anemia in sows

**DOI:** 10.1186/s40104-026-01443-6

**Published:** 2026-06-25

**Authors:** Lingling Sun, Nan Chen, Yunbo Yang, Rui Chen, Yanming Cheng, Yan Chen, Xianghua Yan

**Affiliations:** 1 National Key Laboratory of Agricultural Microbiology, Frontiers Science Center for Animal Breeding and Sustainable Production, Hubei Hongshan Laboratory, College of Animal Sciences and Technology, Huazhong Agricultural University, Wuhan, Hubei 430070 China; 2 National Engineering Research Center for Green Feed and Healthy Breeding, Key Laboratory of Animal Molecular Nutrition, Ministry of Education, Key Laboratory of Animal Nutrition and Feed Science (Eastern of China), Ministry of Agriculture and Rural Affairs, Zhejiang Key Laboratory of Nutrition and Breeding for High-Quality Animal Products Institute of Feed Science, College of Animal Science, Zhejiang University, Hangzhou, Zhejiang 310058 China

**Keywords:** Anemia, Gut microbiota, Hemoglobin, *Kineothrix alysoides*, *Parabacteroides distasonis*, Sows

## Abstract

**Background:**

Sow anemia has been reported to be associated with reduced reproductive performance. Additionally, anemia may alter gut microbiota composition. However, the gut microbiota and metabolic signatures specifically linked to sow anemia status remain poorly understood.

**Results:**

Compared with normal sows, anemic sows had lower hemoglobin levels at gestational days 30, 90, and 110 (*P* < 0.0001). The litter size (16.68 vs. 17.13), live litter size (14.34 vs. 15.13), healthy litter size (13.40 vs. 14.36), and litter weight (18.77 vs. 19.50) of anemic sows were lower than normal sows, but there was no significant difference. Iron transport-related genes showed that placental ferritin expression was significantly higher in normal sows than in anemic sows (*P* = 0.003). Serum antioxidant analysis showed that total antioxidant capacity was significantly lower in anemic sows than in normal sows at gestational days 90 (*P* = 0.026) and 110 (*P* = 0.017). Superoxide dismutase activity was significantly lower in anemic sows than in normal sows at gestational day 90 (*P* = 0.004) and showed a decreasing trend at gestational day 110 (*P* = 0.072). Fecal 16S rRNA analysis revealed distinct gut microbiota compositions between normal and anemic sows. Untargeted metabolomics showed distinct intergroup separation, with significant enrichment of the cytochrome P450 pathway at gestational days 30 and 110 (*P* < 0.05). Furthermore, multiple bacterial taxa showed significant positive correlations with hemoglobin levels, including *Christensenella hongkongensis*, *Gimesia aquarii*, *Shigella dysenteriae*, *Massiliimalia massiliensis*, *Kineothrix alysoides*, *Mediterraneibacter gnavus*, *Parabacteroides distasonis*, and *Treponema* sp.* Marseille-Q3903* (*P* < 0.05).

**Conclusion:**

This study reveals the gut microbial and metabolic characteristics between normal and anemic sows. Two key bacterial species, *P. distasonis* and *K. alysoides*, were identified as associated with anemia status in sows.

**Supplementary Information:**

The online version contains supplementary material available at 10.1186/s40104-026-01443-6.

## Background

Anemia, characterized by a deficiency of red blood cells or low hemoglobin (Hb) concentration, serves as an indicator of nutritional deficiencies or underlying health problems that may affect the health and reproductive performance of sows [1]. Iron is a key trace element for Hb synthesis, as well as for supporting normal embryonic development, antioxidant capacity, and immune function [2]. Iron deficiency arises when bodily iron reserves are depleted, dietary iron intake is insufficient, or iron loss occurs. This impairs Hb production and progresses to iron deficiency anemia (IDA) [3]. IDA is the most prevalent form of anemia in pregnant sows. The gestation period is a critical stage that determines sow reproductive performance, offspring health, and overall swine production efficiency. During this period, rapid fetal development substantially increases the sow’s iron requirements [4]. However, the bioavailability of dietary iron intake fails to meet these elevated physiological demands, resulting in anemia.

Anemia not only compromises sows’ health but also adversely affects reproductive outcomes, thereby reducing overall production efficiency. Furthermore, the Hb levels of piglets are positively correlated with maternal Hb levels before and after farrowing [5]. The farrowing duration is significantly longer in anemic sows than non-anemic sows, which increases the risk of stillbirth [6]. Further research has found that the stillbirth rate in sows is negatively correlated with maternal Hb levels [7]. Therefore, elevating sow Hb levels will likely increase newborn piglet Hb levels and consequently reduce the stillbirth rate during farrowing. Anemia in sows can reduce milk production. Lactoferrin, a milk protein, promotes iron absorption and erythropoiesis [8–10]. Consequently, a reduction in milk output decreases lactoferrin content, leading to insufficient iron in milk [11] and subsequently impairing piglet growth and development. Therefore, gestational Hb levels are directly related to maternal iron reserves and nutritional supply capacity, representing a key factor influencing sow reproductive performance.

Iron supplementation is a common strategy for anemia prevention. Although sow diets typically contain adequate iron in the premix, recent studies on iron supplementation have reported inconsistent efficacy outcomes. Iron supplementation can enhance sow antioxidant capacity and improve piglets iron nutrition, potentially by increasing the availability of lactoferrin and heme iron in milk [12]. However, dietary iron supplementation fails to improve sow reproductive performance and may instead increase fecal iron excretion [13]. The NRC (2012) [14] recommends a dietary iron supplementation level of 80 mg/kg for pregnant sows. The inherent iron content in conventional feed ingredients is relatively high, consequently, the iron level in basal diet frequently meets or exceeds the nutritional requirements of pigs [15]. However, a large-scale U.S. survey revealed that the dietary iron levels in approximately 4.24 million commercial pigs were 1.3–3.7 times higher than the NRC (2012) standard [16]. Despite this overall iron surplus, the prevalence of IDA on pig farms remains high. Excessive iron can antagonize the absorption of other essential minerals, such as zinc, copper, and manganese, by competing for shared intestinal transporters [17]. This mineral imbalance may further compromise sow health and reproductive performance. Excess iron can also interfere with copper metabolism, reduce ceruloplasmin levels, and inhibit zinc absorption [18, 19]. Furthermore, although transferrin can bind zinc and nickel, its binding capacity may be compromised under iron overload conditions. This persistent problem indicates that enhancing iron bioavailability, rather than merely increasing its dietary concentration, is a critical issue requiring resolution.

The gut microbiota, comprising trillions of microorganisms residing in the gastrointestinal tract, plays a crucial role in host metabolism, immune regulation, and nutrient absorption [20]. Gut microbiota imbalance is associated with various metabolic disorders, including anemia [21]. Furthermore, the gut microbiota biosynthesizes and regulates metabolites, including short-chain fatty acids (SCFAs) [22], lactic acid [23], and vitamins, which are crucial for iron metabolism and erythropoiesis. Studies in germ-free rats demonstrate that the absence of live gut microbiota significantly reduces intestinal iron absorption and disrupts systemic iron homeostasis, ultimately leading to anemia [24].

Given the close relationship among gut microbiota, anemia, and iron metabolism, this study systematically characterized the gut microbial communities and untargeted fecal metabolomic profiles of normal and anemic sows across different gestational stages. The objectives were to investigate the impact of anemia on sow production and to identify key microbial taxa and metabolites associated with anemia status in sows.

## Methods

### Animals and experimental design

All animal procedures were reviewed and approved by the Institutional Animal Care and Use Committee of Huazhong Agricultural University (Approval No. HZAUSW-2024-0051). All efforts were made to minimize animal suffering. A total of 194 multiparous Landrace × Yorkshire sows with similar genetic backgrounds were used in this study. The parity distribution was as follows: parity 2 (*n* = 29), parity 3 (*n* = 21), parity 4 (*n* = 20), parity 5 (*n* = 68), and parity ≥ 6 (*n* = 56). The sows were housed and fed in gestation stalls at Jiade Sow Farm (Fusui County, Chongzuo City, Guangxi, China) from May to October 2024. Throughout the experiment, sows were maintained under consistent environmental conditions. All semen used for the sows came from Duroc boars. Sows were fed twice daily (at 7:00 and 14:30). The daily feed allowance was 2.4 kg per sow from gestational days 0 to 85 and was increased to 3.4 kg per sow from day 86 until farrowing. Sows had ad libitum access to water throughout gestation. All diets were formulated according to the National Research Council (NRC, 2012) sow nutrition standards [14]. The composition and nutrient levels of the basal diets for gestation and lactation sows are shown in Tables S1 and S2. The gestation and lactation diets contained 14% and 17% crude protein and 220 mg/kg and 260 mg/kg iron, respectively. Seven days before their expected farrowing date, the sows were moved to the farrowing house, where they received the lactation diet.

The Hb levels of 194 sows were measured at gestational days 30 (30 d), 90 (90 d), and 110 (110 d). A sterile steel lancet was used to puncture the tail vein, and a 10 µL blood sample was collected with a pipette. Hb concentration was immediately measured using a portable hemoglobin meter (Aikang, Hangzhou, China) with compatible test strips, and the results were recorded. All measurements were performed at the same time of day. Each measurement was repeated at least twice, and the average value was used for data analysis. Gestational anemia was defined according to the T/CPPC 1047—2022 standard: Hb < 120 g/L at 30 d, and Hb < 110 g/L at 90 d and 110 d. This standard was developed by the Institute of Subtropical Agriculture, Chinese Academy of Sciences. Applying these criteria to the 194 sows yielded the following classifications: at 30 d, 51 normal and 143 anemic sows; at 90 d, 42 normal and 152 anemic sows; and at 110 d, 72 normal and 122 anemic sows.

### Samples and reproductive performance data collection

Fecal samples were collected from 194 sows at 30 d, 90 d and 110 d using the rectal stimulation method. The procedure was as follows: the operator wore disposable sterile PE gloves on both hands, inserted the index and middle fingers of the right hand into the rectum, and scooped out the feces. If no feces were present in the rectum, gentle stimulation was applied to induce defecation. The collected feces were immediately transferred into sterile 5 mL centrifuge tubes. Gloves were changed after each sow to prevent cross-contamination. After collection, all samples were immediately frozen in liquid nitrogen and stored at −80 °C.

At 30 d, 90 d, and 110 d, 10 sows from each group (normal and anemic) were selected for blood sampling from the anterior vena cava for biochemical testing. Blood collection was performed as follows: a rope was used to hold the sow’s upper jaw, raising the head to approximately a 45° angle. The sampling site was disinfected with an iodine-soaked cotton ball. A disposable blood collection needle was inserted vertically into the anterior vena cava, and approximately 5 mL of blood was withdrawn. Blood samples were allowed to clot at room temperature for 1 h, then centrifuged at 1,250 × *g* for 15 min to obtain the serum supernatant. Serum samples were transferred to 1.5 mL microcentrifuge tubes, immediately frozen in liquid nitrogen, and stored at −80 °C until analysis.

Following farrowing, reproductive performance data were recorded, including litter size, live litter size, healthy litter size (birth weight > 0.6 kg), weak litter size (birth weight ≤ 0.6 kg), mummy fetus, stillbirth, deformed fetus, and litter weight.

Following the method described by Mou et al. [25], placental samples were collected immediately after birth from the piglet whose body weight was closest to the litter average. Sampling was performed at a site opposite the umbilical cord to avoid interference from major blood vessels. The samples were rinsed with 0.9% sodium chloride solution, minced with scissors, and immediately aliquoted into 5 mL centrifuge tubes. The samples were then snap-frozen in liquid nitrogen and stored at −80 °C until analysis.

### Assessment of gene expression

Gene expression levels were detected using reverse transcription polymerase chain reaction (RT-PCR) as previously described by Hu et al. [26]. Total RNA was extracted from the placenta of pigs using Trizol reagent (ABclonal, China). High-quality total RNA (1 μg) was reverse-transcribed into cDNA using ABScript Neo RT Master Mix for qPCR with gDNA Remover (ABclonal, China). The sequences of all primers used are shown in Table S3. qPCR amplification was performed on a Bio-Rad CFX384 Touch qPCR System (Bio-Rad) using 2 × Universal SYBR Green Fast qPCR Mix (ABclonal, China) and gene-specific primer pairs. The qPCR program consisted of an initial denaturation at 95 °C for 3 min, followed by 40 cycles of denaturation at 95 °C for 5 s and annealing at 60 °C for 30 s. Melting curves were generated from 65 °C to 95 °C. Relative gene expression levels were calculated using the 2^−ΔΔCT^ method, with β-actin as the reference gene.

### Determination of antioxidant indicators

The serum antioxidant indicators were measured using commercial kits (Jiancheng, Nanjing, China). The optical density (OD) value was measured using a microplate reader (SuPerMax 3100, Shanpu, Shanghai, China). Malondialdehyde (MDA) (Kit No. A003-1-2) was assayed using the thiobarbituric acid method. The reaction was carried out in a 95 °C water bath. The OD value was measured at 532 nm, and the results were expressed in nmol/mL. Total antioxidant capacity (T-AOC) (Kit No. A015-1-2) was determined using a colorimetric assay. Antioxidants in the serum sample reduce Fe^3+^ to Fe^2+^. The resulting Fe^2+^ forms a stable complex with phenanthroline, and the absorbance of the colored complex, measured at 520 nm, is proportional to the T-AOC. The results were expressed in nmol/L. In the glutathione peroxidase (GSH-Px) (Kit No. A005-1-2) assay, the reaction with dithionitrobenzoic acid generates the 5-mercapto-2-nitrobenzoic acid anion, which produces a stable yellow color. The OD value was measured at 412 nm, and the results were expressed in U/mL. Superoxide dismutase (SOD) (Kit No. A001-3-2) was measured using the WST assay. The OD value was measured at 450 nm, and the result were expressed in U/mL. The intra-assay and inter-assay coefficients of variation (CV%) were as follows: MDA, 2.30% and 5.34%; T-AOC, 3.60% and 6.40%; GSH-Px, 3.56% and 6.80%; SOD, 5.50% and 3.32%.

### Microbial genomic DNA extraction and 16S rRNA gene sequencing

For 16S rRNA sequencing, a subset of sows was selected using a stricter criterion: anemic sows with Hb < 95 g/L and an equal number of normal sows with the highest Hb levels (numbers: 30 d: 44/44, 90 d: 41/41, 110 d: 34/34).

The bacterial genomic DNA was extracted from fecal samples using the FastPure Stool DNA Isolation Kit (MJYH, shanghai, China) according to manufacturer’s instructions. The DNA extract was checked on 1% agarose gel, and DNA concentration and purity were determined with NanoDrop2000 spectrophotometer (Thermo Fisher Scientific, USA). For bacterial community, the bacterial 16S rRNA genes were amplified using the universal bacterial primers 27F (5′-AGRGTTYGATYMTGGCTCAG-3′) and 1492R (5′-RGYTACCTTGTTACGACTT-3′). The PCR products were purified using the AMPure® PB beads (Pacifc Biosciences, CA, USA) and quantified with Qubit 4.0 (Thermo Fisher Scientific, USA).

Purified products were pooled in equimolar and DNA library was constructed using the SMRTbell prep kit 3.0 (Pacifc Biosciences, CA, USA). Purified SMRTbell libraries were sequenced on the Pacbio Sequel IIe System (Pacifc Biosciences, CA, USA) by Majorbio Bio-Pharm Technology Co., Ltd. (Shanghai, China). High-fidelity (HiFi) reads were obtained from the subreads, generated using circular consensus sequencing via the CCS mode of SMRT Link v11.0. The optimized-HiFi reads were de-noised using DADA2_CCS plugin in the Qiime2 (v 2024.2) pipeline with recommended parameters, which obtains single nucleotide resolution based on error profiles within samples. DADA2_CCS denoised sequences are usually called amplicon sequence variants (ASVs). Taxonomic assignment of ASVs was performed using the Naive bayes consensus taxonomy classifier implemented in Qiime2 and the SILVA 16S rRNA database (v138). Bioinformatic analysis of the fecal microbiota was carried out using the Majorbio Cloud platform (https://cloud.majorbio.com). Taxonomic biomarkers were identified using linear discriminant analysis effect size (LEfSe) with screening thresholds of linear discriminant analysis (LDA) > 2 and *P* < 0.05.

In this study, bacterial taxonomy was assigned using the SILVA database (v138). Taxa that could not be reliably classified at the genus level were designated using the prefix ‘norank_’ followed by the lowest confident taxonomic level (e.g., *norank_o_Bacteroidales* for an unclassified taxon at the order level). All taxonomic names are presented in italics following standard nomenclature guidelines.

### Untargeted metabolomic profiling and data analysis

Untargeted metabolomics was performed on the identical sow subset selected for 16S rRNA sequencing. Metabolite extraction followed earlier methods [27]. Briefly, 50 mg of fecal sample was added to a 2-mL centrifuge tube, along with a 6-mm diameter grinding bead, followed by 400 μL of extraction solution (methanol:water = 4:1, v:v) containing four internal standards (e.g., 0.02 mg/mL L-2-chlorophenylalanine). Samples were ground by the Wonbio-96c frozen tissue grinder (Shanghai Wanbo Biotechnology Co., Ltd., China) for 6 min (−10 °C, 50 Hz), followed by low-temperature ultrasonic extraction for 30 min (5 °C, 40 kHz). The samples were left at −20 °C for 30 min, centrifuged for 15 min (4 °C, 13,000 × *g*), and the supernatant was transferred to the injection vial for LC–MS/MS analysis. The LC–MS/MS analysis of sample was conducted on a UHPLC-Orbitrap Exploris 240 system. Mass spectrometry analysis was done in positive and negative ion modes. A quality control (QC) sample was prepared to by mixing equal volumes of all samples, and was tested every 15 samples to monitor the stability of the analysis. Raw data was performed by Progenesis QI v.3.0 (Waters Corporation, Milford, USA) for baseline filtering, peak identification, integration, retention time correction, peak alignment, etc. The metabolites were annotated by searching database, and the main databases were the HMDB, Metlin and Majorbio Database (MJDB) of Majorbio Biotechnology Co., Ltd. (Shanghai, China). The data matrix obtained by searching database was uploaded to the Majorbio cloud platform (https://cloud.majorbio.com) for data analysis. Metabolites were considered differential if they met thresholds: Fold Change (FC) > 1.2 or < 0.833 and *P* < 0.05. Differential metabolites among two groups were mapped into their biochemical pathways through metabolic enrichment and pathway analysis based on KEGG database (http://www.genome.jp/kegg/).

### Quantification of SCFAs

SCFAs in feces were quantified using gas chromatography, as previously described [28]. Briefly, 1 g of feces was weighed, dissolved, and homogenized in 1 mL of methanol. Then, the supernatant was obtained by centrifuging at 12,000 × *g* for 10 min at 4 °C. Subsequently, 25% metaphosphoric acid (4:1, v/v) was added, and the mixture was incubated overnight at 4 °C. Finally, the supernatant was obtained by centrifugation at 12,000 × *g* for 10 min at 4 °C, filtered through a 0.22-μm filter, and analyzed for SCFAs using gas chromatography (GC Trace 1300, Thermo Fisher Scientific, USA).

### Statistical analysis

Statistical analyses and data visualization were performed using SPSS (version 27.0.1), GraphPad Prism (version 9.0.0) and R (version 4.4.2). Normality of data was assessed using the Shapiro–Wilk test. The reproductive performance of sows was modeled using the following general linear model:

$${Y}_{i}=\mu +{\alpha}_{g\left(i\right)}+{\beta}_{p\left(i\right)}+{\varepsilon}_{i}$$

where ${Y}_{i}$ is the reproductive performance of the sow, $\mu$ is the overall mean, ${\alpha}_{g\left(i\right)}$ is the fixed effect of group, ${\beta}_{p\left(i\right)}$ is the fixed effect of parity, and ${\varepsilon}_{i}$ is the residual error. The same model was used to analyze the expression levels of placental ferritin and *TfR1* genes, where ${Y}_{i}$ represents the measured expression level of the ferritin or *TfR1*. Group was modeled as a main effect, and parity was included as a fixed effect. Serum antioxidant markers and SCFAs measured at three time points were analyzed using the following mixed model:

$${Y}_{ij}=\mu +{T}_{j}+{G}_{ij}+{\left(T\times G\right)}_{ij}+{P}_{i}+{u}_{i}+{\varepsilon}_{ij}$$

where ${Y}_{ij}$ is the observation of the dependent variable, $\mu$ is the overall mean, ${T}_{j}$ is the fixed effect of time, ${G}_{ij}$ is the fixed effect of group, ${\left(T\times G\right)}_{ij}$ is the interaction effect between time and group, ${P}_{i}$ is the fixed effect of parity, ${u}_{i}$ is the random effect of sow, and ${\varepsilon}_{ij}$ is the residual error. Beta diversity was calculated using Bray–Curtis distances, and the effect of group was tested using PERMANOVA with parity included as a fixed effect. Differential species were further identified using LEfSe, with LDA > 2 and *P* < 0.05 as the significance thresholds. Metabolites were considered differential if they met the following thresholds: FC > 1.2 or < 0.833, and *P* < 0.05. Correlations among differential bacteria, metabolites, hemoglobin levels, reproductive performance, and SCFAs were calculated by using Spearman’s correlation analysis. Data are presented as mean ± standard error of the mean (SEM). All results were considered significant at *P* < 0.05 and were considered a tendency at *P* ≤ 0.10.

## Results

### Changes in Hb of sows during gestation

**Fig. 1:**
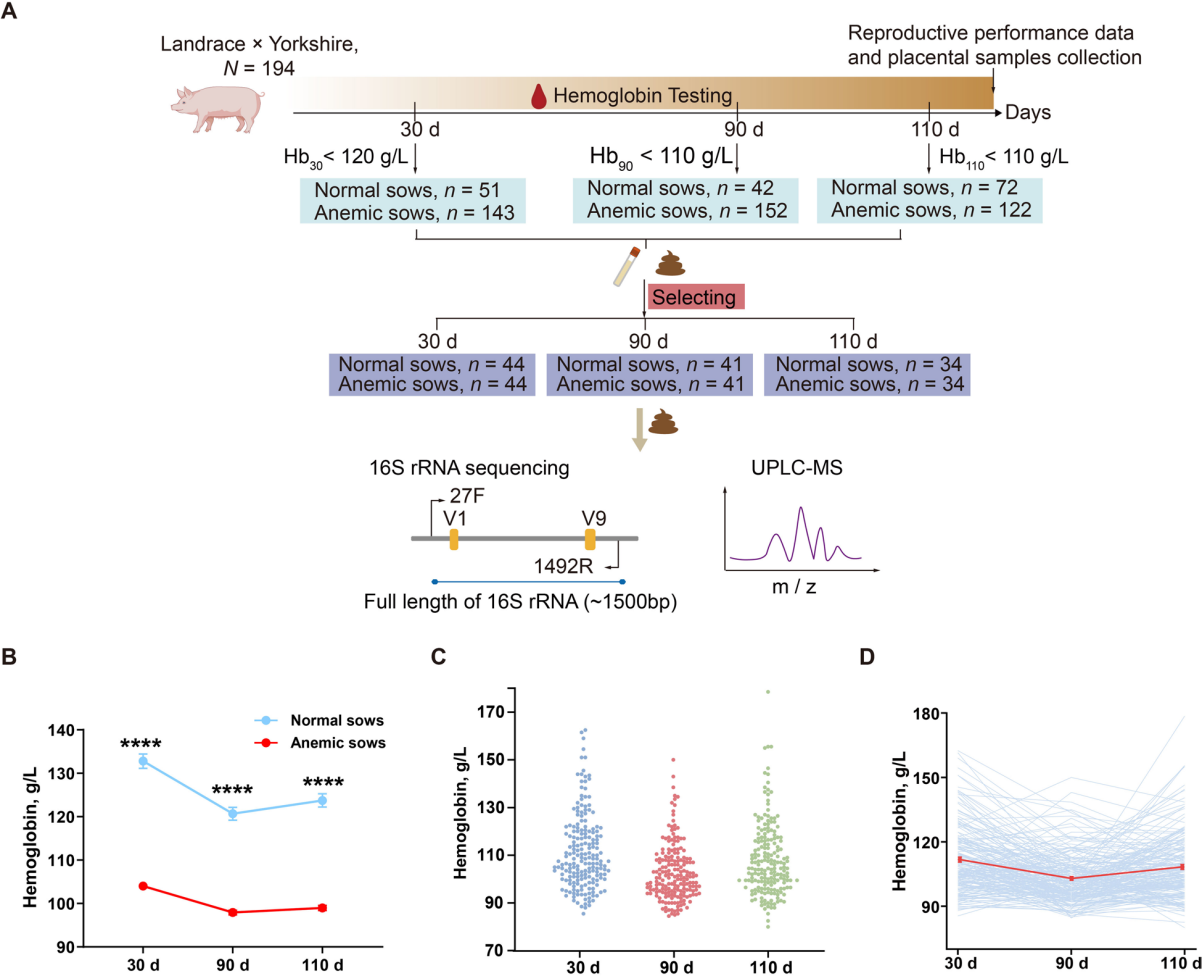
Changes in Hb levels of sows at 30 d, 90 d and 110 d. **A** Flowchart of animal experiments. A total of 194 sows were screened for Hb levels at 30 d, 90 d, and 110 d. Based on Hb thresholds, sows were classified as normal or anemic at each time point (30 d: normal sows = 51, anemic sows = 143; 90 d: normal sows = 42, anemic sows = 152; 110 d: normal sows = 72, anemic sows = 122). For 16S rRNA sequencing and untargeted metabolomics, a subset of sows was selected using a stricter criterion: anemic sows with Hb < 95 g/L, and an equal number of normal sows with the highest Hb levels (numbers: 30 d: 44/44, 90 d: 41/41, 110 d: 34/34). **B** Comparison of hemoglobin levels between normal and anemic sows. **C** Distribution of Hb in sows at 30 d, 90 d and 110 d. **D** Hb dynamics over time. Superimposed on the plots are the estimated mean Hb levels over the study period. The data are expressed as the mean ± SEM. ^****^P < 0.0001. Hb: hemoglobin; 30 d: gestational day 30; 90 d: gestational day 90; 110 d: gestational day 110

Based on the predefined anemia criteria, sows were classified as normal or anemic after Hb testing (Fig. 1A). Hb levels were significantly lower in anemic sows than in normal sows (*P* < 0.0001) (Fig. 1B). The highest and lowest recorded sow Hb levels across the three time points were 178.5 g/L and 80 g/L, respectively (Fig. 1C). At the individual level, sow Hb levels exhibited dynamic changes throughout gestation. The average Hb level was 111.59 g/L at 30 d, then continuously decreased throughout gestation, reaching a low of 102.84 g/L at 90 d. Interestingly, Hb levels increased to 108.16 g/L at 110 d (Fig. 1D).

### Reproductive performance and expression of genes associated with placental iron transport between normal and anemic sows

Anemic sows exhibited lower litter size, live litter size, healthy litter size, and litter weight, but higher weak litter size, stillbirth, and deformed fetus than normal sows, however, these differences were not significant (Table 1). Ferritin and transferrin receptor 1 (TfR1) are key proteins involved in placental iron transport. Ferritin mRNA expression was significantly upregulated in normal sows compared to anemic sows (*P* = 0.003), and *TfR1* mRNA expression was also upregulated, but the difference was not statistically significant (Table 2).

**Table 1:** The reproductive performance of normal and anemic sows

Items	Normal sows (*n* = 72)	Anemic sows (*n* = 122)	*P*-values
Litter size, n	17.13 ± 0.47	16.68 ± 0.37	0.625
Live litter size, n	15.13 ± 0.46	14.34 ± 0.33	0.248
Healthy litter size^1^, n	14.36 ± 0.43	13.40 ± 0.30	0.106
Litter weight, kg	19.50 ± 0.61	18.77 ± 0.46	0.557
Weak litter size^2^, n	0.76 ± 0.09	0.93 ± 0.10	0.238
Stillbirth, n	1.36 ± 0.22	1.66 ± 0.18	0.345
Mummy fetus, n	0.42 ± 0.10	0.38 ± 0.07	0.738
Deformed fetus, n	0.22 ± 0.05	0.30 ± 0.05	0.254

Values are presented as mean ± SEM

^1^Healthy litter size: birth weight > 0.6 kg

^2^Weak litter size: birth weight ≤ 0.6 kg

**Table 2:** The placental gene expression levels in normal and anemic sows

Items	Normal sows (*n* = 8)	Anemic sows (*n* = 8)	*P*-values
Ferritin	0.99 ± 0.57	0.41 ± 0.04	0.003
*TfR1*	1.20 ± 0.28	1.05 ± 0.19	0.776

Values are presented as mean ± SEM

*TfR1* Transferrin receptor 1

### Antioxidant capacity in normal versus anemic sows

Serum antioxidant capacity analysis revealed that T-AOC was significantly lower in anemic sows than in normal sows at 90 d (*P* = 0.026) and 110 d (Table 3, *P* = 0.017). SOD activity was significantly lower in anemic sows than in normal sows at 90 d (*P* = 0.004) and showed a decreasing trend at 110 d (Table 3, *P* = 0.072). No significant differences were observed in the activities of GSH-Px and MDA between the two groups at any time point.

**Table 3:** The antioxidant indicators of normal and anemic sows

Items	Time	Normal sows (*n* = 10)	Anemic sows (*n* = 10)	*P*-values
T-AOC, mmol/L	30 d	0.37 ± 0.03	0.36 ± 0.04	0.853
	90 d	0.43 ± 0.02	0.32 ± 0.01	0.026
	110 d	0.44 ± 0.01	0.35 ± 0.02	0.017
SOD, U/mL	30 d	88.76 ± 0.77	88.73 ± 1.16	0.452
	90 d	92.10 ± 0.78	88.08 ± 1.37	0.004
	110 d	96.70 ± 1.37	95.46 ± 1.15	0.072
GSH-Px, U/mL	30 d	1752.07 ± 59.29	1751.35 ± 60.63	0.841
	90 d	1595.85 ± 60.58	1,471.27 ± 42.79	0.775
	110 d	1764.86 ± 74.00	1718.48 ± 65.48	0.922
MDA, nmol/mL	30 d	1.55 ± 0.09	1.41 ± 0.13	0.710
	90 d	2.25 ± 0.28	2.19 ± 0.23	0.862
	110 d	1.78 ± 0.11	2.04 ± 0.18	0.425

Values are presented as mean ± SEM

*30 d* Gestational day 30, *90 d* Gestational day 90, *110 d* Gestational day 110, *MDA* Malonaldehyde, *SOD* Superoxide dismutase, *GSH-Px* Glutathione peroxidase, *T-AOC* Total antioxidant capacity

### The diversity and structure of gut microbiota between normal and anemic sows

**Fig. 2:**
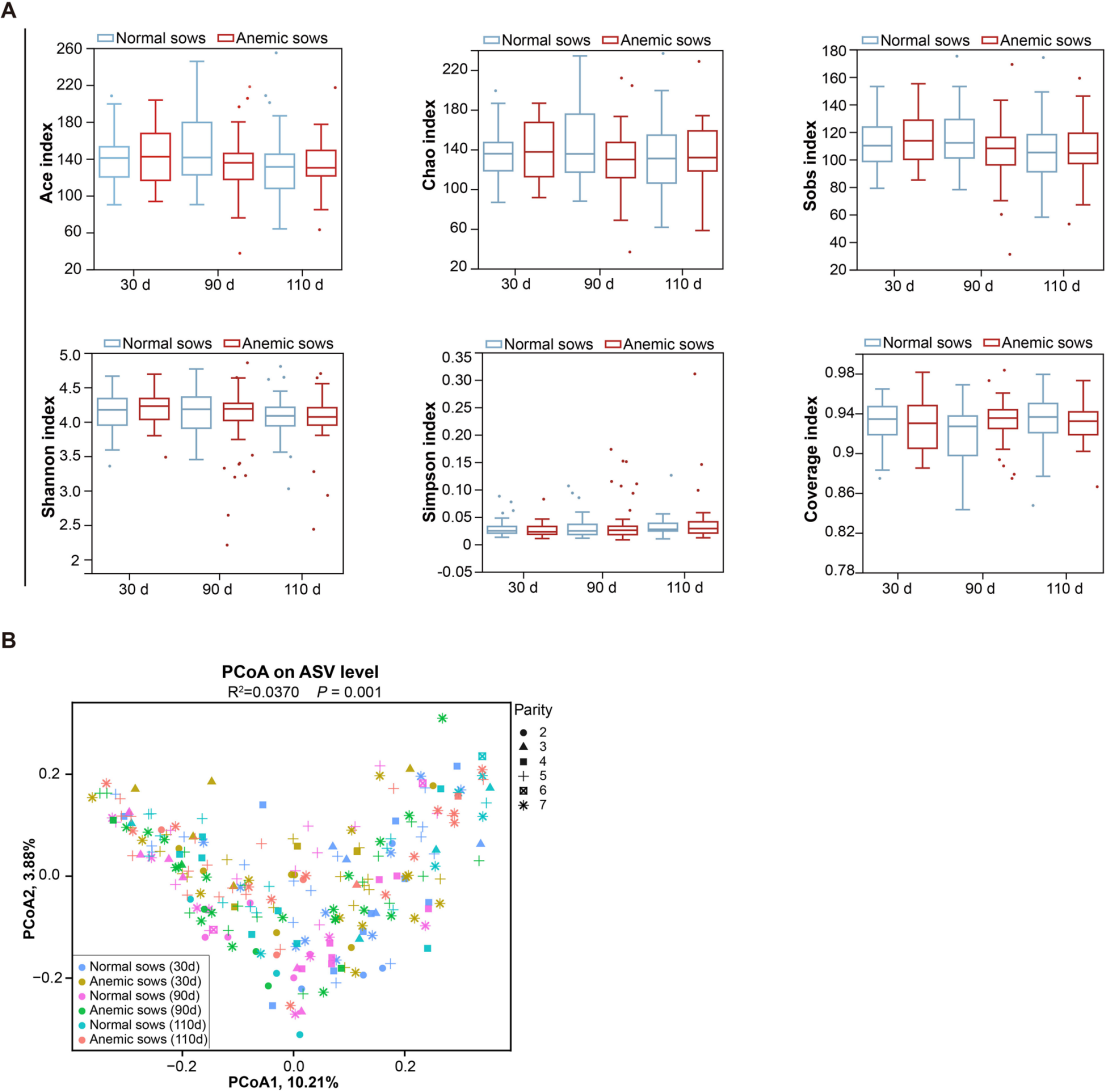
Comparison of the diversity and structure of gut microbiota between normal and anemic sows. **A** Alpha diversity indices of gut microbiota including ACE index, Chao index, Sobs index, Shannon index, Simpson index and Coverage index. **B** Beta diversity presented by PCoA based on Bray–Curtis distances. 30 d: gestational day 30; 90 d: gestational day 90; 110 d: gestational day 110

16S rRNA sequencing of feces was performed at 30 d, 90 d and 110 d (Fig. 1A). The alpha diversity indexes including ACE index, Chao index, Sobs index, Shannon index, Simpson index, and Coverage index were not significantly different between normal and anemic sows (Fig. 2A), indicating that gut microbiota diversity and richness were similar between the two groups across time points. PCoA based on Bray–Curtis distances showed significant differences in fecal microbial composition between normal and anemic sows at all three time points (Fig. 2B).

**Fig. 3:**
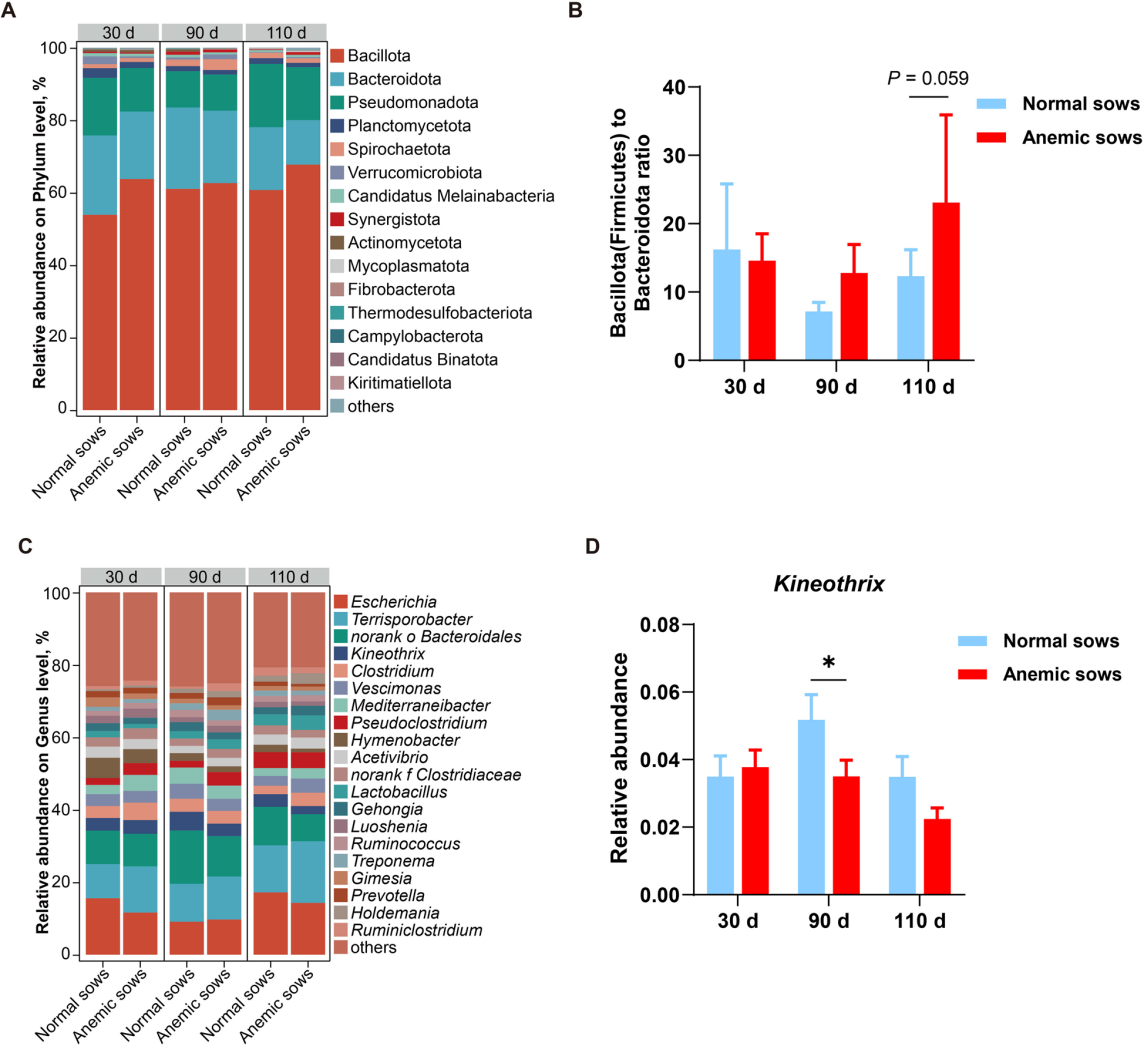
Gut microbial composition of normal and anemic sows. **A** and **C** Comparison of the major microbes at the phylum (**A**) and genus(**C**) levels between normal and anemic sows respectively. **B** The Bacillota (Firmicutes) to Bacteroidota ratio of normal and anemic sows at 30 d, 90 d and 110 d. **D** The relative abundance of *Kineothrix* in normal and anemic sows at 30 d, 90 d and 110 d. 30 d: gestational day 30; 90 d: gestational day 90; 110 d: gestational day 110. ^*^P < 0.05

At the phylum level, Bacillota (previously Firmicutes, 61.6%), Bacteroidota (18.8%), and Pseudomonadota (13.3%) dominated the microbial composition across all three time points. Compared with normal sows, anemic sows showed a higher proportion of Bacillota (previously Firmicutes) and a lower proportion of Bacteroidota (Fig. 3A). At 110 d, normal sows exhibited a decreasing trend in the Bacillota (previously Firmicutes)-to-Bacteroidota (F/B) ratio compared with anemic sows (*P* = 0.059, Fig. 3B). At the genus level, *Escherichia* (34.6%) and *Terrisporobacter* (12.4%) were the two dominant genera in both groups. An unclassified operational taxonomic unit within the order Bacteroidales, provisionally named *norank_o_Bacteroidales*, accounted for 10.4% of the total community in both groups (Fig. 3C). The relative abundance of *Kineothrix* was higher in normal sows than in anemic sows at 90 d and 110 d, with a significant difference observed at 90 d (*P* < 0.05, Fig. 3D).

### The marker bacteria between normal and anemic sows

**Fig. 4:**
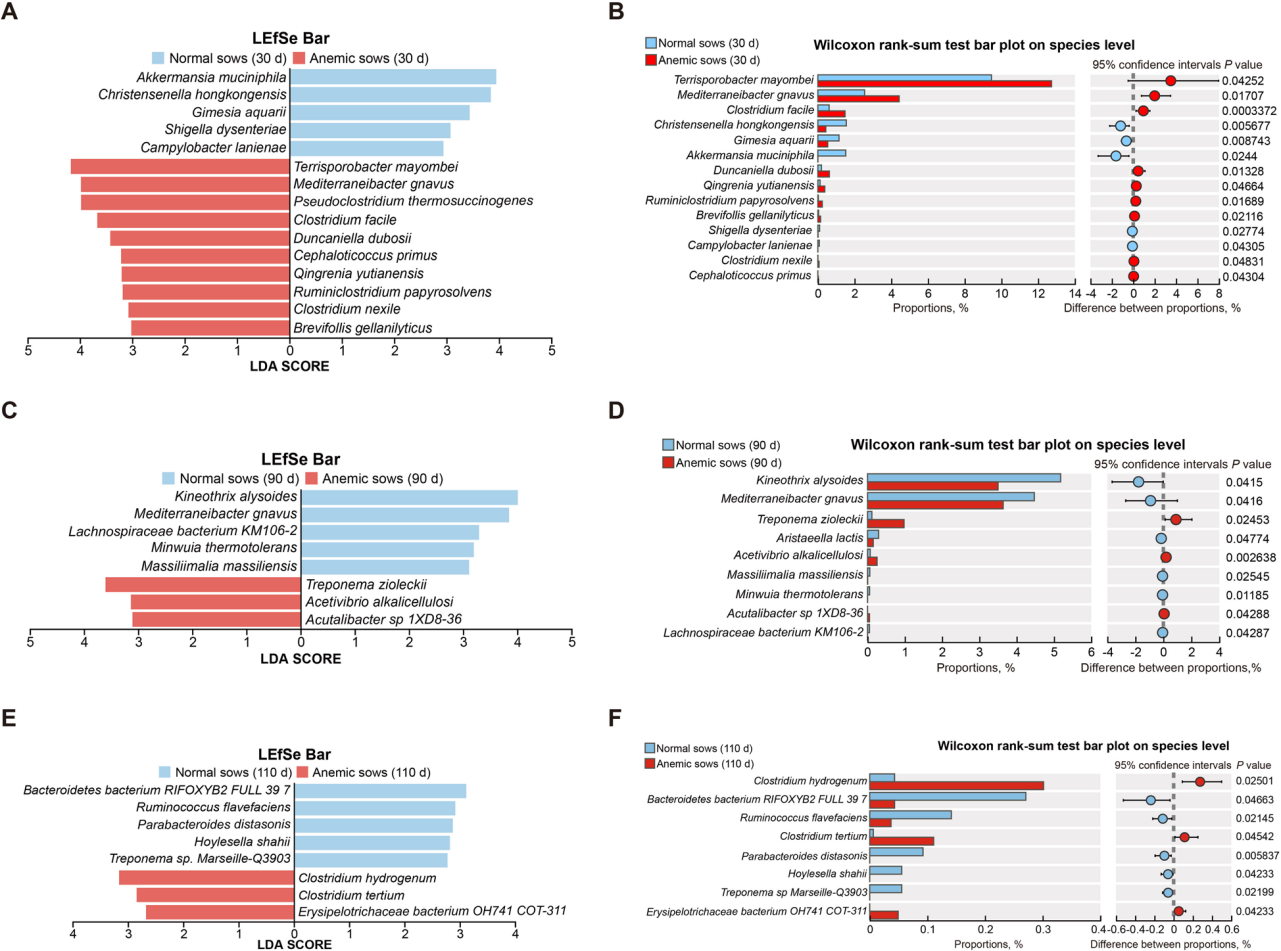
The marker bacteria between normal and anemic sows at 30 d, 90 d and 110 d. The histogram of linear discriminant analysis (LDA) score revealed the most differentially abundant taxa at the species level between normal and anemic sows at 30 d (**A**), 90 d (**C**), and 110 d (**E**). The proportion of differential bacteria at 30 d (**B**), 90 d (**D**), and 110 d (**F**). 30 d: gestational day 30; 90 d: gestational day 90; 110 d: gestational day 110

The LDA score histogram revealed marker bacteria at the species level across the three time points (Fig. 4). At 30 d, five differentially abundant bacteria were identified in normal sows: *Akkermansia muciniphila*, *Christensenella hongkongensis*, *Gimesia aquarii*, *Shigella dysenteriae*, and *Campylobacter lanienae* (*P* < 0.05, Fig. 4A and B). At 90 d, *Kineothrix alysoides*, *Mediterraneibacter gnavus*, *Lachnospiraceae bacterium KM106-2*, *Minwuia thermotolerans*, and *Massiliimalia massiliensis* were identified as differentially abundant bacteria in normal sows (*P* < 0.05, Fig. 4C and D). At 110 d, normal sows contained more *Bacteroidetes bacterium RIFOXYB2 FULL 39 7*, *Hoylesella shahii*, *Parabacteroides distasonis*, *Ruminococcus flavefaciens*, and *Treponema* sp. *Marseille-Q3903* than anemic sows (*P* < 0.05, Fig. 4E and F).

### The concentrations of SCFAs

Fecal SCFAs analysis showed that normal sows had significantly higher butyrate concentrations than anemic sows at 90 d (Table 4, *P* = 0.047). At 110 d, anemic sows showed a trend toward elevated levels of butyrate (*P* = 0.053) and isovalerate (Table 4, *P* = 0.068). Microbial analysis revealed that the relative abundance of *K. alysoides* at 90 d was significantly higher in normal sows than that in anemic sows (*P* < 0.05, Fig. 4D). *K. alysoides*, a known saccharolytic butyrate-producing bacterium [29, 30], was consistent with the significant increase in fecal butyrate levels observed in normal sows at 90 d (Table 4).

**Table 4:** The SCFAs content of normal and anemic sows

Items	Time	Normal sows	Anemic sows	*P*-values
Acetate, µmol/g	30 d	27.39 ± 1.17	28.00 ± 1.54	0.648
	90 d	31.78 ± 1.19	30.43 ± 1.26	0.342
	110 d	29.63 ± 1.40	31.26 ± 1.39	0.458
Propionate, µmol/g	30 d	19.00 ± 1.24	17.54 ± 1.00	0.452
	90 d	24.19 ± 1.35	21.80 ± 1.06	0.140
	110 d	23.37 ± 1.53	25.84 ± 1.62	0.196
Butyrate, µmol/g	30 d	6.95 ± 0.53	7.18 ± 0.63	0.769
	90 d	10.13 ± 0.58	8.75 ± 0.57	0.047
	110 d	9.94 ± 0.81	11.94 ± 0.99	0.053
Isobutyrate, µmol/g	30 d	2.63 ± 0.14	2.59 ± 0.16	0.822
	90 d	2.82 ± 0.12	3.15 ± 0.43	0.306
	110 d	2.89 ± 0.13	3.35 ± 0.27	0.193
Valerate, µmol/g	30 d	1.69 ± 0.11	1.65 ± 0.13	0.803
	90 d	1.98 ± 0.10	1.92 ± 0.12	0.518
	110 d	2.04 ± 0.13	2.40 ± 0.20	0.133
Isovalerate, µmol/g	30 d	3.98 ± 0.21	3.98 ± 0.25	0.970
	90 d	4.33 ± 0.19	4.20 ± 0.24	0.650
	110 d	4.38 ± 0.22	5.11 ± 0.39	0.068

Values are presented as mean ± SEM

The number of sows per group at each time point: 30 d: *n* = 44, 90 d:* n* = 41, 110 d:* n* = 34

*30 d* Gestational day 30, *90 d* Gestational day 90, *110 d* Gestational day 110

### Metabolomic profiling between normal and anemic sows

**Fig. 5:**
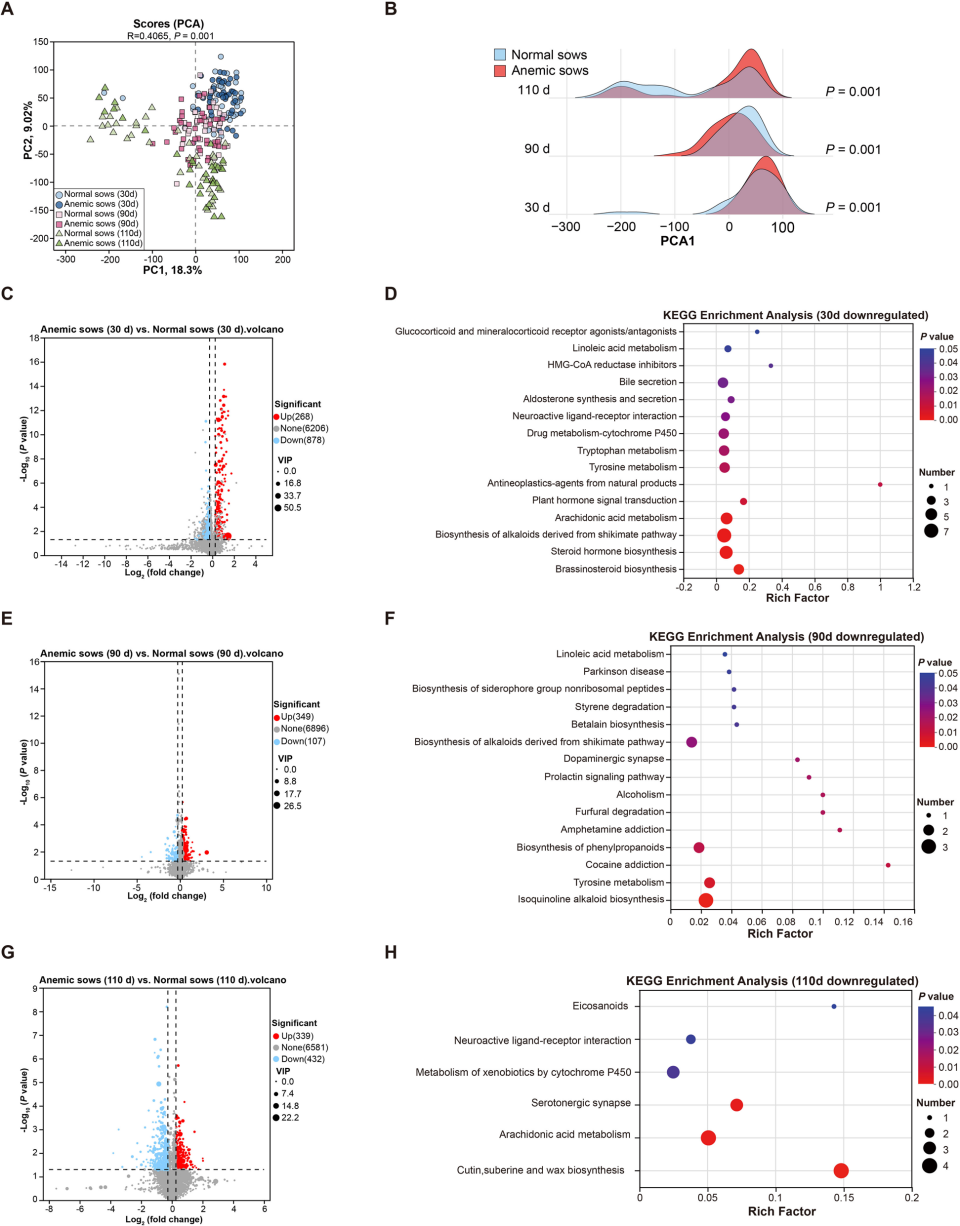
Metabolite profiles between normal and anemic sows at 30 d, 90 d, and 110 d. **A** Principal component analysis (PCA) plots of metabolic profiles of normal and anemic sows at 30 d, 90 d, and 110 d. **B** The density plot shows a comparison of the PCA1 of normal and anemic sows at 30 d, 90 d, and 110 d. Volcano plot illustrating differentially abundant metabolites between normal and anemic sows at 30 d (**C**), 90 d (**E**), and 110 d (**G**). KEGG enrichment pathways (*P* < 0.05) of differential downregulated metabolites in normal and anemic sows at 30 d (**D**), 90 d (**F**), and 110 d (**H**). 30 d: gestational day 30; 90 d: gestational day 90; 110 d: gestational day 110

Untargeted metabolomics analysis of fecal samples was conducted to elucidate differences in microbial metabolites between normal and anemic sows. Principal component analysis (PCA) revealed that PC1 and PC2 accounted for 18.3% and 9.02% of variance, respectively, with significant differences in fecal metabolic profiles between groups (*P* < 0.001, Fig. 5A). The metabolic profiles gradually shifted toward the left side of the PCA axis over time, with significant differences between normal and anemic sows at each time point (*P* < 0.001, Fig. 5B). Volcano plots showed metabolites significantly upregulated and downregulated metabolites at 30 d (Fig. 5C), 90 d (Fig. 5E), and 110 d (Fig. 5G). KEGG pathway analysis was performed using downregulated metabolites to investigate their potential biological functions at each time point. At 30 d, fifteen pathways were significantly enriched (*P* < 0.05), including brassinosteroid biosynthesis, steroid hormone biosynthesis, biosynthesis of alkaloids derived from shikimate pathway, arachidonic acid metabolism, and plant hormone signal transduction, etc. (Fig. 5D), with 43 metabolites enriched across these pathways (Table S4). At 90 d, metabolites were primarily enriched in fifteen pathways (*P* < 0.05), including isoquinoline alkaloid biosynthesis, tyrosine metabolism, cocaine addiction, biosynthesis of phenylpropanoids, and amphetamine addiction, etc. (Fig. 5F), with nine metabolites enriched in these pathways (Table S5). At 110 d, six pathways significantly enriched (*P* < 0.05), including cutin, suberine, and wax biosynthesis, arachidonic acid metabolism, serotonergic synapse, metabolism of xenobiotics by cytochrome P450, neuroactive ligand-receptor interaction, and eicosanoids at 110 d (Fig. 5H), with twelve metabolites enriched in these pathways (Table S6).

### Correlation analysis of gut microbiota and metabolites related to Hb levels, reproductive performance, and SCFAs

**Fig. 6:**
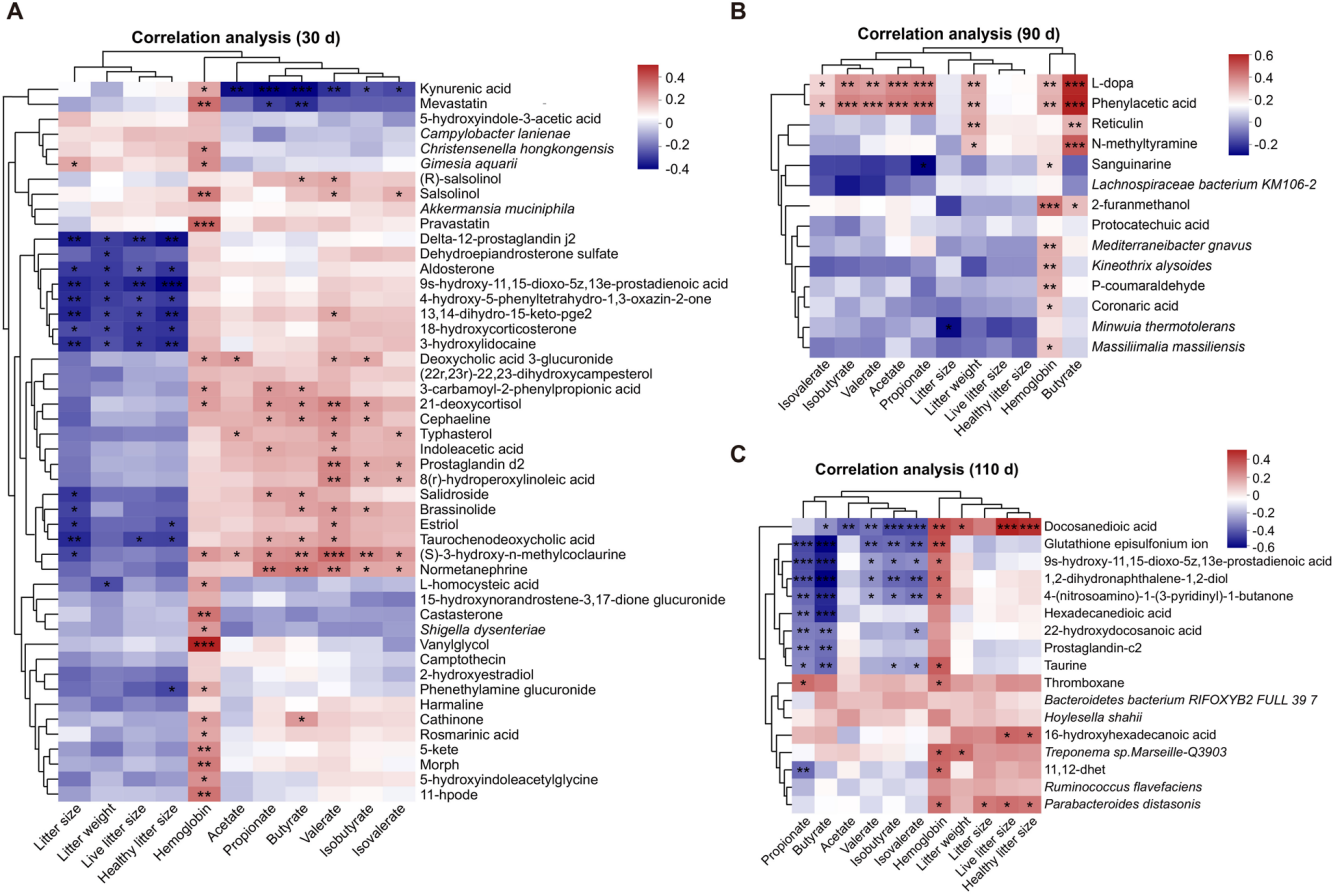
Correlation analysis of intestinal microbiota and metabolites related to Hb levels, reproductive performance, and SCFAs. **A** Correlation analysis between marker bacteria and 43 differential metabolites with Hb levels, reproductive performance, and SCFAs at 30 d. **B** Correlation analysis between marker bacteria and 9 differential metabolites with Hb levels, reproductive performance, and SCFAs at 90 d. **C** Correlation analysis between marker bacteria and 12 differential metabolites with Hb levels, reproductive performance, and SCFAs at 110 d. The colors range from blue (negative correlation) to red (positive correlation). ^*^P < 0.05, ^**^P < 0.01, and ^***^P < 0.001. 30 d: gestational day 30; 90 d: gestational day 90; 110 d: gestational day 110; Hb: hemoglobin; SCFAs: short-chain fatty acids

As shown in Fig. 6, at 30 d, 18 metabolites, including kynurenic acid, mevastatin, salsolinol, pravastatin, deoxycholic acid 3-glucuronide, 3-carbamoyl-2-phenylpropionic acid, 21-deoxycortisol, (S)-3-hydroxy-n-methylcoclaurine, L-homocysteic acid, castasterone, vanylglycol, phenethylamine glucuronide, cathinone, rosmarinic acid, 5-kete, morph, 5-hydroxyindoleacetylglycine, and 11-hpode were significantly positively correlated with Hb levels. Significant positive correlations were observed between microbes and Hb levels, including *C. hongkongensis*, *G. aquarii*, and *S. dysenteriae*. Fifteen differential metabolites showed significant negative correlations with reproductive performance (litter size, live litter size, healthy litter size, and litter weight). Eighteen differential metabolites were significantly positively correlated with SCFAs. In contrast, kynurenic acid and mevastatin were significantly negatively correlated with SCFAs (Fig. 6A). At 90 d, metabolites including L-dopa, phenylacetic acid, sanguinarine, 2-furanmethanol, p-coumaraldehyde, and coronaric acid showed significant positive correlations with Hb levels. Significant positive correlations were also observed between microbes, including *M. massiliensis*, *K. alysoides*, and *M. gnavus*, and Hb levels. L-Dopa, phenylacetic acid, reticulin, N-methyltyramine, and 2-furanmethanol exhibited significant positive associations with SCFAs, while only sanguinarine was negatively correlated with propionate. Regarding reproductive performance, L-dopa, phenylacetic acid, reticulin, and N-methyltyramine showed a positive correlation with litter weight. *M. thermotolerans* was significantly negatively associated with litter size (Fig. 6B). At 110 d, significant positive correlations were observed between Hb levels and multiple metabolites, including docosanedioic acid, glutathione episulfonium ion, 9s-hydroxy-11,15-dioxo-5z,13e-prostadienoic acid, 1,2-dihydronaphthalene-1,2-diol, 4-(nitrosoamino)-1-(3-pyridinyl)-1-butanone, taurine, thromboxane, 11,12-dhet. *P. distasonis* and *Treponema* sp.* Marseille-Q3903* were also significantly positively correlated with Hb levels. Ten differential metabolites exhibited significant negative correlations with SCFAs, while thromboxane was the only metabolite that showed a significant positive correlation specifically with propionate. For reproductive performance, docosanedioic acid was significantly positively correlated with litter weight, live litter size, and healthy litter size, while 16-hydroxyhexadecanoic acid showed a significant positive association specifically with live litter size and healthy litter size. Among microbes, *P. distasonis* showed a significant positive association with litter size, live litter size, and healthy litter size. *Treponema* sp.* Marseille-Q3903* demonstrated a significant positive correlation with litter weight (Fig. 6C).

## Discussion

Currently, research on the effects of sow anemia on reproductive performance and gut microbiota remains limited. This study conducted large-scale monitoring and analysis of Hb levels and reproductive performance in pregnant sows. Unlike previous studies that focused only on individual reproductive performance such as farrowing duration, stillbirth, the present study provided a more comprehensive analysis by integrating longitudinal Hb monitoring with a broader range of performance outcomes.

In this study, Hb levels declined between 30 d and 90 d and then partially recovered by 110 d, consistent with previous findings [31]. As gestation progresses, fetal body weight and nutritional requirements increase exponentially. Beginning in mid-to-late gestation, the sow’s total blood volume increases significantly to support the rapid growth of the uterus, placenta, mammary glands, and fetus. However, the rate of plasma expansion far exceeds that of red blood cell production [32], leading to a decline in the blood’s oxygen-carrying capacity. This, in turn, triggers increased erythropoietin secretion by the kidneys, which stimulates the bone marrow to accelerate red blood cell production [33]. Notably, Hb levels were lowest at 90 d, a critical period for rapid fetal growth and placental maturation. During this stage, maternal iron mobilization is insufficient to meet increased fetal iron demands, thereby exacerbating systemic iron deficiency. As a key substrate for Hb biosynthesis, increased dietary iron intake enhances the supply of raw materials for erythropoiesis [34]. Starting seven days before farrowing, sows received a lactation diet containing 260 mg/kg iron, which was higher than the 220 mg/kg iron provided in the gestation diets at 30 d and 90 d. Thus, the rise in Hb levels at 110 d may be attributed to the higher iron content in the lactation diet compensating for increased iron consumption, however, this requires further verification.

Reproductive performance in sows is a core element of sustainable swine industry development, directly affecting production efficiency, economic benefits, and food security [35–37]. The farrowing duration in anemic sows is twice as long as that in non-anemic sows, and prepartum anemia may lead to adverse outcomes for both sows and piglets [6]. Furthermore, Bhattarai et al. [38] demonstrated a positive correlation between maternal and piglet Hb levels and a negative correlation between maternal Hb levels and stillbirth rate. Our results showed that normal sows had higher reproductive performance than anemic sows, however, these differences were not statistically significant. Parity was included as a fixed effect in our statistical model to control for its known influence on reproductive outcomes. However, other unmeasured factors, including nutritional status and genetic background, may have contributed to the lack of significance. Consequently, future studies with more standardized experimental designs are warranted to accurately evaluate the impact of maternal anemia on reproductive performance. During late gestation, the placenta plays a pivotal role in iron transport to meet the substantial demands of rapidly growing fetuses [39]. Ferritin is a crucial iron storage protein that reflects systemic iron status. In human placental cells, abundant ferritin functions as a key iron transport carrier, mediating maternal–fetal iron transfer through specific receptor pathway. IDA in pregnant mothers leads to decreased placental ferritin levels, potentially impairing transplacental iron transport. Ferritin expression progressively increases throughout gestation, peaking at term. This peak corresponds to the period of maximal fetal iron acquisition, which supports accelerated growth during late gestation [40, 41]. Upregulation of placental ferritin mRNA expression in normal sows indicated enhanced iron storage capacity, which may facilitate more efficient iron transfer to the fetus compared with anemic sows.

Anemia is closely associated with iron status, and systemic iron levels influence antioxidant capacity. Iron plays a key role in the body’s enzymatic antioxidant system. During normal metabolism, the breakdown of superoxide anions generates byproducts such as hydrogen peroxide and other organic peroxides. Iron effectively neutralizes these reactive species, thereby ensuring the proper function of catalytic reactions within the antioxidant enzyme network [42]. Specifically, T-AOC comprehensively evaluates antioxidant status in animals, whereas GSH-Px and SOD activities reflect free radical dynamics and tissue oxidative damage [43, 44]. MDA, a highly toxic lipid peroxidation metabolite, indicates the extent of lipid peroxidation in liver tissue. Our results showed that normal sows exhibited higher T-AOC and SOD activity levels at 90 d and 110 d. Notably, inadequate iron intake increases stress sensitivity and reduces antioxidant capacity in animals [45], which is particularly relevant to pregnant sows. During late gestation, rapid fetal growth, increased maternal energy intake, and accelerated maternal metabolism collectively enhance reactive oxygen species (ROS) production. Moreover, the elevated rates of digestion, absorption, and tissue mobilization required to support fetal and mammary development further exacerbate ROS generation [46, 47]. Our findings suggested that maternal anemia during gestation primarily impairs T-AOC and SOD-mediated redox regulation, rather than inducing widespread lipid peroxidation or disrupting glutathione-dependent antioxidant pathways. The maternal antioxidant system declines in late gestation, characterized by reduced plasma antioxidant enzyme activity and T-AOC, thereby potentially predisposing anemic sows to oxidative stress.

To date, most microbiome studies on anemia have focused on humans, with few investigations into gut microbiota differences between normal and anemic sows. Our work combined metabolomics with fecal microbiome analysis for the first time to explore the relationships among gut microbiota, metabolic profiles, and Hb levels in pregnant sows. The ratio of F/B reflects gut microbiota balance and serves as an indicator of overall host health status [48]. The increased F/B ratio observed in anemic sows in the present study indicated gut microbiota dysbiosis, consistent with observations in anemic rat models [49]. We found that, at the genus level, the relative abundance of *Kineothrix* at 90 d was significantly higher in normal sows than in anemic sows *Kineothrix* has been documented as a dietary fiber-fermenting bacterium capable of producing butyrate and other SCFAs, which influence host energy metabolism, immune regulation, and intestinal barrier function [29, 50]. SCFAs have been reported to mediate iron absorption [51]. Specifically, the gut microbiota of infants with IDA exhibits a reduced relative abundance of butyrate-producing bacteria such as *Butyricicoccus*, *Coprococcus*, and *Roseburia* [52]. Dostal et al. [53] similarly demonstrated a significant reduction in butyrate content in iron-deficient rats, with potential benefits of butyrate for intestinal health, including anti-inflammatory and antioxidant activity [54]. Gas chromatography revealed that fecal butyrate concentration was significantly higher in normal sows than in anemic sows at 90 d, consistent with the observed enrichment of the butyrate-producing genus *Kineothrix* in the normal group at the same time point. Collectively, these findings indicated that SCFAs, particularly butyrate, may be closely associated with anemia status and warrant further investigation.

Gut microbiota homeostasis is critical for regulating gut metabolism. Comprehensive untargeted metabolomic profiling was performed on fecal samples from normal and anemic sows. KEGG pathway enrichment analysis revealed that the differential metabolites were enriched in neuroactive ligand-receptor interaction, linoleic acid metabolism, cytochrome P450 metabolism, arachidonic acid metabolism, steroid hormone biosynthesis, and bile secretion, etc. Notably, the differential metabolites were enriched in the cytochrome P450 metabolic pathway at both 30 d and 110 d. Cytochrome P450 enzyme is a membrane-bound heme protein that plays key roles in detoxifying exogenous substances, cellular metabolism, and maintaining homeostasis [55]. Heme, a complex composed of iron ions and porphyrin rings, serves as a cofactor for various hemoglobin proteins and is responsible for oxygen binding and transport [56]. Iron deficiency may impair hemoglobin synthesis, thereby disrupting the function of the cytochrome P450 metabolic pathway. These findings suggest that the observed alterations in the cytochrome P450 metabolic pathway may be related to anemia. Correlation analysis between the potential key gut microbes and Hb levels across the three time points revealed that *C. hongkongensis*, *G. aquarii*, *S. dysenteriae*, *M. massiliensis*, *K. alysoides*, *M. gnavus*, *P. distasonis*, and *Treponema* sp.* Marseille-Q3903* were positively correlated with Hb levels in normal sows. The relative abundance of *P. distasonis* was significantly higher in normal sows than in anemic sows at 110 d. As a core gut bacterium, the abundance of *P. distasonis* inversely correlates with the prevalence of several human diseases, including inflammatory bowel disease, multiple sclerosis, nonalcoholic fatty liver disease, and obesity [57–59]. Zhang et al. [60] reported that the abundances of *Parabacteroides* sp. CT06 and *P. distasonis* were significantly upregulated in the feces of non-anemic patients and were positively correlated with 1,25-dihydroxyvitamin D_3_ levels. Notably, iron supplementation has been shown to increase serum 25-dihydroxyvitamin D_3_ concentrations in children [61], and a study on children with IDA also revealed a correlation between 25-dihydroxyvitamin D_3_ levels and iron metabolism [62]. *Parabacteroides* can metabolize bile acids to produce lithocholic acid, a potent agonist of vitamin D receptor (VDR) [63]. Vitamin D has also been shown to directly inhibit hepcidin expression through VDR signaling and promote iron release by upregulating ferritin, thereby facilitating Hb synthesis [60]. Collectively, these findings suggested that *P. distasonis* may be associated with anemia.

Although our study provided correlational data on Hb levels, fecal microbiota, and metabolites in sows, several limitations should be noted. First, Hb levels reportedly decrease with increasing parity. As this study used multiparous sows without controlling for parity, it remains unclear whether the observed Hb differences are attributable solely to parity variation or involve interactions with gut microbiota, metabolites, and other factors. Future research should control for parity by evaluating Hb levels in sows of the same parity. Second, iron absorption occurs primarily in the proximal intestine, yet this study focused on fecal microbiota and metabolites. Fecal samples may not fully capture microbial and metabolic activity at the proximal intestinal site, which limits mechanistic interpretation of microbiota-iron absorption links. Furthermore, the dietary transition from gestation to lactation feed, which contains higher iron levels at 110 d, represents an important confounding factor. We speculate that this dietary shift could transiently alter luminal iron availability, thereby promoting an increase in the abundance of iron-associated *P. distasonis* and *R. flavefaciens* [64, 65] at 110 d and affecting SCFAs [65] or cytochrome P450 metabolic pathways. Such diet-induced microbial and metabolic fluctuations may partly explain the unique microbiota and metabolite profiles observed at 110 d. Therefore, future studies should adopt standardized dietary protocols and integrate gut microbiota analysis with tissue metabolomics. This integrated approach will yield deeper insights into the relationships between the microbiome, metabolites, and sow Hb levels, as well as the underlying physiological mechanisms.

## Conclusions

In summary, this study revealed that normal sows exhibit higher antioxidant capacity and higher placental ferritin gene expression, and it elucidated the gut microbial and metabolic signatures associated with anemia in pregnant sows. Through correlation analysis, we identified *P. distasonis* and *K. alysoides* as key bacterial species potentially associated with anemia status. Further research is needed to clarify their specific roles and mechanisms in host iron metabolism and anemia. Overall, these findings provide novel insights into the relationship between sow anemia and gut microbiota and lay a foundation for the future development of microbiota-targeted strategies to promote sow health.

## Supplementary Information

Additional file 1: Table S1. Composition and nutrient levels of the gestation diets. Table S2. Composition and nutrient levels of the lactation diets. Table S3. Primer sequences used for qPCR. Table S4. Differential metabolites (30 d). Table S5. Differential metabolites (90 d). Table S6. Differential metabolites (110 d).

## Data Availability

All data generated or analyzed during this study can be made available by the corresponding author upon reasonable request.

## Abbreviations

ASVs: Amplicon sequence variants
GSH-Px: Glutathione peroxidase
Hb: Hemoglobin
IDA: Iron deficiency anemia
LDA: Linear discriminant analysis
MDA: Malonaldehyde
OD: Optical density
PCA: Principal component analysis
ROS: Reactive oxygen species
SCFAs: Short-chain fatty acids
SOD: Superoxide dismutase
T-AOC: Total antioxidant capacity
TfR: Transferrin receptor
TfR1: Transferrin receptor 1
VDR: Vitamin D receptor
30 d: Gestational day 30
90 d: Gestational day 90
110 d: Gestational day 110
