# Supplementary material for: Multi-omics analysis reveals Parabacteroides distasonis and Kineothrix alysoides as potential key bacterial species associated with anemia in sows
Source: J Anim Sci Biotechnol. 2026 Jun 25;17:129. doi: 10.1186/s40104-026-01443-6 (PMC13295343; doi:10.1186/s40104-026-01443-6)
Supplement: Supplementary file 1 — Additional file 1: Table S1. Composition and nutrient levels of the gestation diets. Table S2. Composition and nutrient levels of the lactation diets. Table S3. Primer sequences used for qPCR. Table S4. Differential metabolites (30 d). Table S5. Differential metabolites (90 d). Table S6. Differential metabolites (110 d). [file 40104_2026_1443_MOESM1_ESM.docx]

**Supplementary Tables**

**Table S1**. Composition and nutrient levels of the gestation diets

| Item | Gestation diet |
| --- | --- |
| Ingredients (%) |  |
| Corn | 51 |
| Soybean meal | 15 |
| Soybean hulls | 13 |
| Bran | 10 |
| Beet pulp | 6.0 |
| Glucose | 1.0 |
| Premix^1^ | 4.0 |
| Total | 100 |
| Calculated nutrient composition |  |
| Net energy (MJ/kg) | 9.21 |
| Crude protein (%) | 14 |
| Crude fiber (%) | 9.5 |
| Neutral detergent fiber (%) | 20 |
| Calcium (%) | 0.85 |
| Available phosphorus (%) | 0.35 |
| Digestible lysine (%) | 0.65 |
| Digestible threonine (%) | 0.49 |
| Digestible methionine (%) | 0.18 |
| Digestible methionine + cysteine (%) | 0.46 |
| Digestible tryptophan (%) | 0.13 |
| Digestible valine (%) | 0.49 |

^1^Premix provided vitamins and minerals per kilogram of diets: vitamin A 11,500 IU, vitamin D_3_ 3,500 IU, vitamin E 120 IU, vitamin B_1_ 3.4 mg, vitamin B_2_ 9.8 mg, vitamin B_6_ 5.8 mg, vitamin B_12_ 0.034 mg, niacin 30 mg, folic Acid 4.8 mg, d-pantothenic acid 20 mg, iron 220 mg, copper 18 mg, zinc 120 mg, manganese 60 mg, iodine 0.4 mg, selenium 0.2 mg and chromium 0.2 mg.

**Table S2**. Composition and nutrient levels of the lactation diets

| Item | Lactation diet |
| --- | --- |
| Ingredients (%) |  |
| Corn | 50 |
| Flour | 12.5 |
| Soybean meal | 9.0 |
| Puffed soybeans | 8.0 |
| Fermented soybean meal | 4.0 |
| Bran | 4.0 |
| Beet pulp | 2.0 |
| Soybean oil | 2.5 |
| Fish meal | 3.0 |
| Glucose | 1.0 |
| Premix^1^ | 4.0 |
| Total | 100 |
| Calculated nutrient composition |  |
| Net energy (MJ/kg) | 10.42 |
| Crude protein (%) | 17 |
| Crude fiber (%) | 4.0 |
| Neutral detergent fiber (%) | 12 |
| Calcium (%) | 0.90 |
| Available phosphorus (%) | 0.35 |
| Digestible lysine (%) | 1.0 |
| Digestible threonine (%) | 0.66 |
| Digestible methionine (%) | 0.3 |
| Digestible methionine + cysteine (%) | 0.6 |
| Digestible tryptophan (%) | 0.19 |
| Digestible valine (%) | 0.85 |

^1^Premix provided vitamins and minerals per kilogram of diets: vitamin A 15,000 IU, vitamin D_3_ 4,000 IU, vitamin E 200 IU, vitamin B_1_ 4.0 mg, vitamin B_2_ 12 mg, vitamin B_6_ 8.0 mg, vitamin B_12_ 0.04 mg, niacin 40 mg, folic Acid 5.0 mg, d-pantothenic acid 30 mg, iron 260 mg, copper 20 mg, zinc 140 mg, manganese 70 mg, iodine 0.5 mg, selenium 0.3 mg and chromium 0.2 mg.

**Table S3**. Primer sequences used for qPCR

| Primers | Forward (5’ to 3’) | Reverse (5’ to 3’) |
| --- | --- | --- |
| *β-actin* | ACACGGTGCCCATCTACGAG | GCTTCTCCTTGATGTCCCGC |
| *TfR1* | AGTGGCTGTATTCTGCTCGTG | CGGAGATACATAGGGTGAAAG |
| Ferritin | CAACGAGGTGCCCGAATCTT | GCGTCTCAATGAAGTCACAC |

**Table S4**. Differential metabolites (30 d)

| **Metabolic pathways** | **Metabolites** | ***P* value** |
| --- | --- | --- |
| Brassinosteroid biosynthesis | Brassinolide; typhasterol; castasterone;  (22r,23r)-22,23-dihydroxycampesterol | 0.0004 |
| Steroid hormone biosynthesis | Estriol; 2-hydroxyestradiol;  21-deoxycortisol; 18-hydroxycorticosterone;  dehydroepiandrosterone sulfate; aldosterone | 0.0013 |
| Biosynthesis of alkaloids derived from shikimate pathway | (R)-salsolinol; morph; harmaline; cathinone; cephaeline; camptothecin;  (s)-3-hydroxy-n-methylcoclaurine; salsolinol | 0.0019 |
| Arachidonic acid metabolism | 9s-hydroxy-11,15-dioxo-5z,13e-prostadienoic acid; prostaglandin d2; 5-kete;  delta-12-prostaglandin j2;  13,14-dihydro-15-keto-pge2 | 0.0028 |
| Plant hormone signal transduction | Brassinolide; indoleacetic acid | 0.0091 |
| Antineoplastics - agents from natural products | Camptothecin | 0.0123 |
| Tyrosine metabolism | Normetanephrine; salidroside;  rosmarinic acid; vanylglycol | 0.0153 |
| Tryptophan metabolism | 5-hydroxyindole-3-acetic acid;  5-hydroxyindoleacetylglycine;  indoleacetic acid; kynurenic acid | 0.0188 |
| Drug metabolism - cytochrome P450 | 3-carbamoyl-2-phenylpropionic acid;  Morph; 3-hydroxylidocaine;  4-hydroxy-5-phenyltetrahydro-1,3-oxazin-2-one | 0.0220 |
| Neuroactive ligand-receptor interaction | Morph; prostaglandin d2;  l-homocysteic acid | 0.0271 |
| Aldosterone synthesis and secretion | Aldosterone; 18-hydroxycorticosterone | 0.0295 |
| Bile secretion | Phenethylamine glucuronide; taurochenodeoxycholic acid; dehydroepiandrosterone sulfate; pravastatin; deoxycholic acid 3-glucuronide;  15-hydroxynorandrostene-3,17-dione glucuronide | 0.0312 |
| HMG-CoA reductase inhibitors | Mevastatin | 0.0365 |
| Linoleic acid metabolism | 11-hpode; 8(r)-hydroperoxylinoleic acid | 0.0461 |
| Glucocorticoid and mineralocorticoid receptor agonists/antagonists | Aldosterone | 0.0484 |

**Table S5**. Differential metabolites (90 d)

| **Metabolic pathways** | **Metabolites** | ***P* value** |
| --- | --- | --- |
| Isoquinoline alkaloid biosynthesis | Sanguinarine; l-dopa; reticulin | 0.0011 |
| Tyrosine metabolism | L-dopa; n-methyltyramine | 0.0073 |
| Cocaine addiction | L-dopa | 0.0118 |
| Biosynthesis of phenylpropanoids | P-coumaraldehyde; protocatechuic acid | 0.0134 |
| Amphetamine addiction | L-dopa | 0.0152 |
| Furfural degradation | 2-furanmethanol | 0.0168 |
| Alcoholism | L-dopa | 0.0168 |
| Prolactin signaling pathway | L-dopa | 0.0185 |
| Dopaminergic synapse | L-dopa | 0.0202 |
| Biosynthesis of alkaloids derived from shikimate pathway | L-dopa | 0.0235 |
| Betalain biosynthesis | L-dopa | 0.0383 |
| Styrene degradation | Phenylacetic acid | 0.0399 |
| Biosynthesis of siderophore group nonribosomal peptides | Protocatechuic acid | 0.0399 |
| Parkinson disease | L-dopa | 0.0432 |
| Linoleic acid metabolism | Coronaric acid | 0.0464 |

**Table S6**. Differential metabolites (110 d)

| **Metabolic pathways** | **Metabolites** | ***P* value** |
| --- | --- | --- |
| Cutin, suberine and wax biosynthesis | 22-hydroxydocosanoic acid; docosanedioic acid;  16-hydroxyhexadecanoic acid; hexadecanedioic acid | 0.00001 |
| Arachidonic acid metabolism | 9s-hydroxy-11,15-dioxo-5z,13e-prostadienoic acid; 11,12-dhet; prostaglandin-c2; thromboxane | 0.0011 |
| Serotonergic synapse | 11,12-dhet; prostaglandin-c2; thromboxane | 0.0018 |
| Metabolism of xenobiotics by cytochrome P450 | Glutathione episulfonium ion;  1,2-dihydronaphthalene-1,2-diol;  4-(nitrosoamino)-1-(3-pyridinyl)-1-butanone | 0.0331 |
| Neuroactive ligand-receptor interaction | Taurine; thromboxane | 0.0381 |
| Eicosanoids | 9s-hydroxy-11,15-dioxo-5z, 13e-prostadienoic acid; 11,12-dhet; prostaglandin-c2; thromboxane | 0.0403 |
